# Editorial: Ferroptosis: intersections, implications, and innovations in programmed cell death

**DOI:** 10.3389/fcell.2025.1635046

**Published:** 2025-06-17

**Authors:** Bilal Cig, Patrice X. Petit, Juan Carlos Mayo

**Affiliations:** ^1^ Kirsehir Ahi Evran University School of Medicine Physiology Department, Kirsehir, Türkiye; ^2^ SSPIN Saints-Pères Paris Institut de Neurosciences, CNRS UMR 8003, “Mitochondria, Apoptosis and Autophagy Signalling” Université de Paris—Campus Saint-Germain, Paris, France; ^3^ Department of Morphology and Cell Biology, School of Medicine, University of Oviedo, Oviedo, Spain; ^4^ University Institute of Oncology of the Principality of Asturias (IUOPA), Oviedo, Spain; ^5^ Health Research Institute of the Principality of Asturias (ISPA), Avda. University Hospital, Oviedo, Spain

**Keywords:** ferroptosis, ROS - reactive oxygen species, lipid perodixation, GSH, GPx4

Ferroptosis is a series of events characterized by lipid peroxidation and disruptions in iron metabolism. Ferroptosis, first described by Dixon and colleagues in 2012 as a distinct form of regulated cell death, is morphologically and biochemically different from apoptosis, necrosis, and autophagy (Scott et al., 2012).

In this process, cell membrane damage occurs via reactive oxygen species (ROS) and lipoxygenases. Mitochondrial shrinkage, increased membrane density, and loss of crista are hallmarks of the morphological changes observed, as well as biochemical markers such as glutathione (GSH) depletion and decreased GPX4 activity. Iron metabolism imbalance leads to high levels of Fe^2+^ levels, which trigger the Fenton reaction and increases ROS production. In turn, ROS accelerate lipid peroxidation, a process driven primarily by the oxidation of polyunsaturated fatty acids (PUFAs) via lipoxygenases, positioning lipid peroxidation as a key driver of ferroptosis. The primary cellular defense mechanism against ferroptosis is the GSH-GPX4 axis, distruption of which sensitizes cells to ferroptotic death (Stockwell et al., 2017). A better understanding of ferroptosis could lead to new therapeutic strategies for the treatment of diseases such as neurodegenerative diseases, metabolic disorders and cancers.

The first article by Sun et al. reported the role of Tumor protein P73 (TAp73P) in regulating both proliferation and ferroptosis in bovine mammary epithelial cells (BMECs). Using RSL3 to inhibit GPX4 and activate ferroptotic pathways, the study shows that TAp73P suppresses cell proliferation and upregulates ferroptosis-related genes such as TFRC and PTGS2, indicating a dual functional role. In the second study, Xiong et al. present a Fe@Ba nanozyme—assembled with baicalein from traditional Chinese medicine—that effectively neutralizes ROS and suppresses ferroptosis in cisplatin-induced acute kidney injury (AKI). The nanozyme inhibits lipid peroxidation, restores GSH levels, enhances GPX4 expression, and significantly reduces renal damage through its antioxidant capacity. Key ferroptosis-regulating axes include the GSH/GPX4 axis, GCH1/BH4/DHFR axis, and FSP1/CoQ10/NADH axis (Forcina and Dixon, 2019). In the context of non-alcoholic fatty liver disease (NAFLD), ferroptosis is strongly linked with both inflammation and disrupted lipid metabolism. Therapeutic strategies targeting ferroptosis—such as Ferrostatin-1, α-tocopherol, Lip-1, Tβ4, and ginkgolide B—along with lifestyle interventions like exercise and intermittent fasting, have shown anti-inflammatory and hepatoprotective effects (Yang et al., 2020). Li et al. highlight the mechanistic importance of ferroptosis in NAFLD, focusing on its connection to iron dysregulation and lipid peroxidation. Their work demonstrates how exercise activates antioxidant pathways (e.g., NRF2, GPX4, SLC7A11), regulates iron via ferroportin (FPN), and suppresses inflammatory responses via NF-κB and STING signaling, ultimately slowing disease progression.

A review by Zhang et al. comprehensively explores the role of SIRT1, a NAD^+^-dependent histone deacetylase, in redox homeostasis, lipid peroxidation, iron metabolism, and inflammation—core biochemical processes of ferroptosis. Through modulation of pathways such as Nrf2-HO-1, p53-p21, and AMPK, SIRT1 suppresses ferroptosis and exhibits protective effects across multiple disease models, positioning it as a next-generation therapeutic target in disorders ranging from neurodegeneration to diabetes. In another review, Lu et al. underscore ferroptosis as a key contributor to osteoarthritis progression, including chondrocyte loss, synovial inflammation, and subchondral bone remodeling. By focusing on biomarkers like GPX4, TfR1, and NCOA4, the review opens the door for ferroptosis-centered diagnostic and therapeutic approaches in osteoarthritis and suggests future studies should examine combinatory therapies with ferroptosis inhibitors and immunomodulators.

A comprehensive bioinformatics and cell-based study by Ma et al. identified SOX13, a transcription factor belonging to the SOX family, as a potential prognostic biomarker and therapeutic target in ferroptosis-based interventions. In thyroid cancer (THCA) cells, SOX13 modulates ferroptosis-related genes and immune cell infiltration, thereby suppressing tumor progression and immune evasion. Li et al. explore the interaction between ferroptosis and immune response in the context of intervertebral disc degeneration (IDD). They identify seven key ferroptosis-related genes, particularly *MT1G* and *CA9*, that hold promise for diagnostic and therapeutic applications in IDD. Mitochondrial lipid peroxidation plays a crucial but not exclusive role in ferroptosis. He Huan and colleagues demonstrate that mitochondrial lipid oxidation is necessary but not sufficient for ferroptosis unless accompanied by cytosolic ROS accumulation, underscoring a dual-compartmental requirement for this death pathway.

Emerging evidence suggests that ferroptosis contributes to the pathophysiology of renal ischemia-reperfusion injury. In a study by Zhou et al., CD44 was found to be overexpressed at the gene and protein level; its blockade with anti-CD44 therapy attenuated ferroptosis and M1 macrophage infiltration, preserving kidney structure and function. A perspective by Wan Seok Yang assigns a pivotal initiating and regulatory role to the endoplasmic reticulum (ER) membrane in ferroptotic death, proposing it as a future organelle-specific therapeutic target. In a review by Su et al., ferroptosis is highlighted as a dual-edged process—enhancing tumor suppression during radiotherapy while also contributing to radiation-induced damage in healthy tissues. The final review by Liu et al. Focuses on heat shock proteins (HSPs), identifying them as bidirectional regulators of ferroptosis through iron metabolism, GSH/GPX4 signaling, and lipid peroxidation, with therapeutic implications in cancer and neurodegenerative diseases.

Together, the studies in this Research Topic provide in-depth insights into the molecular basis of ferroptosis in various pathological contexts. This information is essential for developing next-generation diagnostic tools and targeted therapeutic strategies with high translational value.
